# Telencephalon Organoids Derived from an Individual with ADHD Show Altered Neurodevelopment of Early Cortical Layer Structure

**DOI:** 10.1007/s12015-023-10519-z

**Published:** 2023-03-06

**Authors:** Danmeng Zhang, Noriomi Eguchi, Satoshi Okazaki, Ichiro Sora, Akitoyo Hishimoto

**Affiliations:** Department of Psychiatry, Kobe University Graduate School of Medicine, Kobe, Japan

**Keywords:** Differentiation, Ventricular zone, Cortical plate, Symmetric cell division, Proliferation, Apoptosis

## Abstract

Attention-deficit/hyperactivity disorder (ADHD) is a neurodevelopmental disorder that occurs in early childhood and can persist to adulthood. It can affect many aspects of a patient's daily life, so it is necessary to explore the mechanism and pathological alterations. For this purpose, we applied induced pluripotent stem cell (iPSC)-derived telencephalon organoids to recapitulate the alterations occurring in the early cerebral cortex of ADHD patients. We found that telencephalon organoids of ADHD showed less growth of layer structures than control-derived organoids. On day 35 of differentiation, the thinner cortex layer structures of ADHD-derived organoids contained more neurons than those of control-derived organoids. Furthermore, ADHD-derived organoids showed a decrease in cell proliferation during development from day 35 to 56. On day 56 of differentiation, there was a significant difference in the proportion of symmetric and asymmetric cell division between the ADHD and control groups. In addition, we observed increased cell apoptosis in ADHD during early development. These results show alterations in the characteristics of neural stem cells and the formation of layer structures, which might indicate key roles in the pathogenesis of ADHD. Our organoids exhibit the cortical developmental alterations observed in neuroimaging studies, providing an experimental foundation for understanding the pathological mechanisms of ADHD.

**Graphical Abstract:**

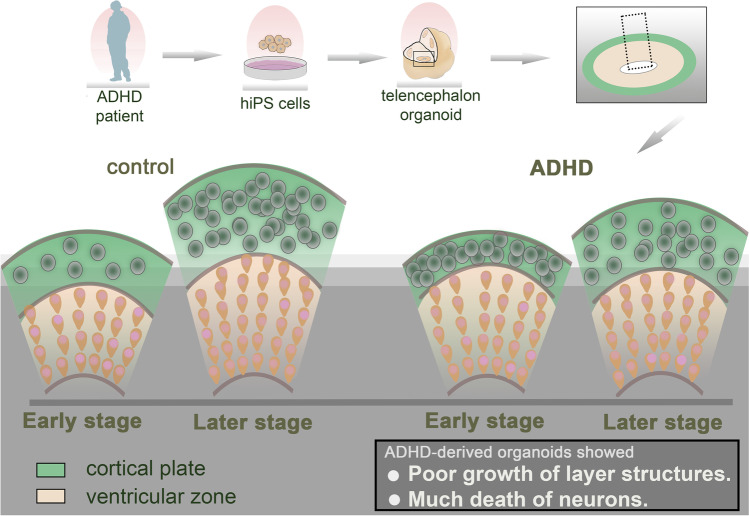

**Supplementary Information:**

The online version contains supplementary material available at 10.1007/s12015-023-10519-z.

## Introduction

Attention-deficit/hyperactivity disorder (ADHD) is a neurodevelopmental disorder mainly characterized by symptoms of inattention, impulsivity, and hyperactivity. The symptoms can be observed from early childhood and often persist to adulthood [1, 2]. This disorder affects 5% of children and adolescents and 2.5% of adults worldwide and can cause failure in life activities such as education and communication throughout the lifetime [3, 4]. ADHD is one of the most heritable neuropsychiatric disorders [5, 6]. Although it has been suggested that common biological or genetic mechanisms exist in ADHD, they are still unclear [2, 7, 8].

The symptoms of ADHD are associated with a combination of clinical, neurodevelopmental and cognitive factors, all of which are based on distinct neuroanatomical foundations [9]. Previous studies have reported alterations in the cerebral cortex of ADHD [10]. Structural changes in the frontal cortex and reduced functional activation play a key role in cognitive processes such as emotion, response inhibition and attention [11, 12]. Symptoms of ADHD can improve over time during maturation and development, and delayed maturation may be related to pathogenesis [13]. It was reported that the surface area of the cerebral cortex showed significant differences between children with ADHD and age-matched controls, but the same was not true of adolescents or adults with ADHD [14–16]. Other studies have mentioned that the prefrontal cortex of children with ADHD showed significantly delayed maturation compared to control [17]. These studies suggest that morphological changes in the cerebral cortex, especially in the development and maturation of the cortex, might play important roles in the pathogenesis of ADHD.

Although prenatal neurodevelopment and postnatal neuroplasticity are considered to be inextricably linked to the pathophysiology of psychiatric disorders, including ADHD [18], poor accessibility often makes it difficult to study them: postmortem brains of people with ADHD are unavailable in many cases, and animal models cannot completely exhibit the phenotypes of ADHD [19, 20]. Recent studies have shown that organoids, the products of three-dimensional tissue culture using induced pluripotent stem cells (iPSCs), can recapitulate the development of human organs (including the brain) in vitro. Brain organoids can mimic the early stage of neurodevelopment and pathology of neuropsychiatric disorders [21, 22], suggesting that they have the potential to reveal the unknown mechanisms of ADHD. Therefore, we used a three-dimensional iPSC culture method known as serum-free floating culture of embryoid body–like aggregates with quick reaggregation (SFEBq) [23] with slight modification [24] to uncover the pathogenesis of ADHD in the early cerebral cortex, especially the telencephalon.

## Materials and Methods

### Generation of Human iPSCs

All studies were performed according to protocols approved by Kobe University. Peripheral blood mononuclear cells were donated by an 18-year-old male patient diagnosed with ADHD with DSM-IV-TR. iPSCs of ADHD were generated from CD34-positive cells in mononuclear cells isolated from peripheral blood as previously described [25]. Control iPSCs were provided by the RIKEN BioResource Research Center (RIKEN BRC). Details of the origin and generation of iPSCs are shown in Table 1. The pluripotency and multipotency of iPSCs derived from an ADHD patient were confirmed by immunocytochemistry (Supplementary Fig. 1). Chromosomes were analyzed by Chromocenter (http://chromocenter.com/) and confirmed that there were no abnormal chromosomes in the iPSCs of the ADHD patient (Supplementary Fig. 2). All iPSCs were maintained as previously described [25].

**Table 1:** Details of the origin and generation of iPSCs

	Cell ID	Age	Sex	Vector	Reprograming factors
Contol-1	409B2	36	female	Episomal vectors	Oct3/4, Sox2, Klf4, L-Myc, Lin28, p53 shRNA
Contol-2	201B7			Retrovirus vectors	Oct3/4, Sox2, Klf4, c-Myc
Contol-3	1383D2	36	Male	Episomal vectors	Oct3/4, Sox2, Klf4, L-Myc, Lin28, TP53 shRNA
ADHD-1	77B4	18	male	Sendai virus vectors	Oct3/4, Sox2, Klf4, c-Myc
ADHD-2	77B9				
ADHD-3	77B11				

### Cortical Tissues Differentiated from Human iPSCs

Cortical differentiation from iPSCs using the modified SFEBq method was performed as described previously [24]. Human iPSCs were dissociated into single cells and then reaggregated using cortical differentiation medium in 96-well V-bottom plates at a density of 5,000 cells per well. Half of the medium was changed every 3–4 days. From day 0 to day 18, the medium contained GMEM, 20% KSR, NEAA, 0.1 mM 2-ME, sodium pyruvate, IWR-1-endo and SB431542, human FGF2 (removed after day 6), and Y-27632 (added on day 3). After day 18, the medium was changed to DMEM/F12 with GlutaMAX-I, N2 supplement, and chemically defined lipid concentrate, and floating cell aggregates were transferred to an EZSPHERE dish. On day 35, 10% FBS, 5 mg/ml heparin, and 1% Matrigel (growth factor reduced) (Corning) were added to the medium (Supplementary Fig. 3).

After 35 or 56 days of differentiation, the tissues were fixed in 4% PFA/PBS for 40 min and cryoprotected in 20% sucrose/PBS overnight at 4 °C. Fixed tissues were embedded in frozen section compound (Leica) and sliced into 10 µm thick sections. The sliced tissues were transferred to CREST-coated glass slides (Matsunami Glass). Slides were washed with PBS, permeabilized with 0.3% Triton X-100 for 10 min, and blocked with 10% normal donkey serum in 0.3% Triton X-100/PBS for 30 min, followed by incubation with primary antibody overnight at 4℃. Primary antibodies against the following antigens were used at the specified dilutions: FOXG1 (TakaRa, 1:1000), TUJ1 (Abcam, 1:1000), SOX2 (Abcam, 1:1000), CTIP2 (Abcam, 1:1000), TBR1 (Abcam, 1:1000; Santa Cruz), phospho-histone H3 (Cell Signaling, 1:500), cleaved caspase 3 (Cell Signaling, 1:200), and Ki67 (BD Pharmingen, 1:200). Fluorescence-tagged secondary antibodies (Jackson) were reacted on the following day. The nuclei were counterstained with DAPI (BD Pharmingen, 1:2000).

### Statistical Analysis

The morphology of sliced tissues was measured using ImageJ (NIH). Cortical structures with diameters less than 200 µm were excluded from measurement. All data are presented as the mean ± standard deviation (S.D.). Statistical analyses were performed using GraphPad Prism software version 9 (GraphPad Software, CA). For multiple comparisons, the Kruskal‒Wallis test was used followed by a post hoc test. The chi-square test was used for contingency table analyses. A p value < 0.05 was considered to be statistically significant.

## Results

### Generation of Telencephalon Organoids

**Fig. 1:**
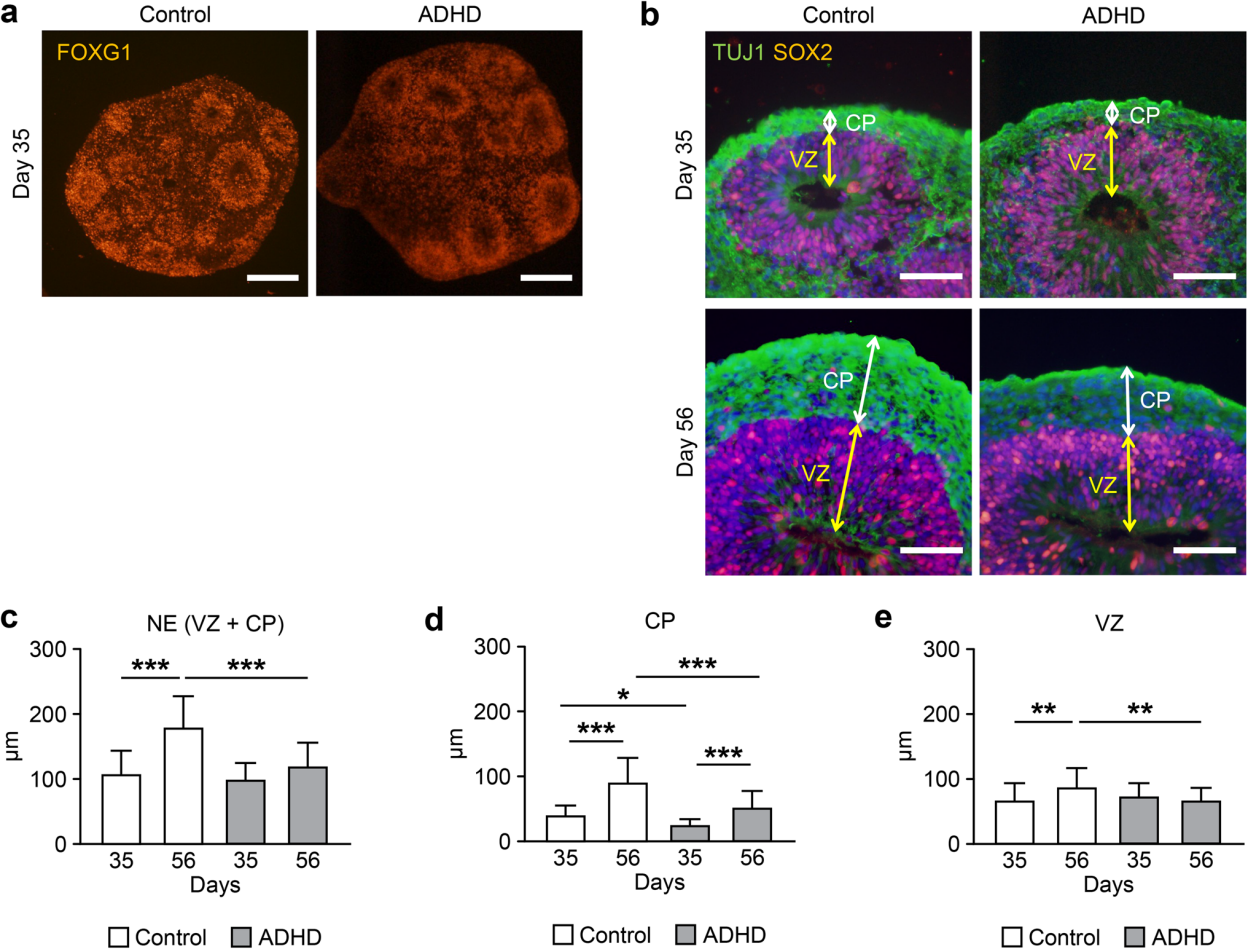
Comparison of layer structures in telencephalon organoids derived from ADHD and control. (**a**) Expression of FOXG1, a telencephalon-specific marker protein, at day 35 of differentiation. Scale bars: 200 μm. (**b**) Layer structures in the organoids at day 35 and 56. Both control-derived and ADHD-derived organoids showed SOX2-expressing neural stem cells in ventricular zone-like structures. The neuron-specific marker protein TUJ1 was expressed in the outer cortical plate–like structure. Scale bars: 40 μm. (**c**) Thickness of neuroepithelium-like structures (NE). (**d**) Thickness of cortical plate–like structures (CP). (**e**) Thickness of ventricular zone–like structures (VZ). Control: day 35 (*n* = 30), day 56 (*n* = 33); ADHD: day 35 (*n* = 30), day 56 (*n* = 41). *P < 0.05, **P < 0.01, ***P < 0.001

Next, we used iPSCs from ADHD and control individuals to generate telencephalon organoids. After 35 days of differentiation (day 35), cell aggregates expressed FOXG1, a specific marker of the telencephalon (Fig. 1a). They contained cell layers expressing TUJ1 and SOX2, indicating layers of neurons and neural stem cells, similar to the cortical plate (CP) and ventricular zone (VZ) observed in the early neurodevelopment of the cerebral cortex (Fig. 1b). These results indicated that they successfully differentiated into telencephalon organoids and recapitulated the early cerebral cortex.

### Thickness of Layer Structures in the Telencephalon Organoids

To distinguish morphological changes occurring in the early cortex in ADHD, we measured the thickness of layer structures in the telencephalon organoids (Fig. 1c). The thickness of the neuroepithelium-like structure (NE), defined as the total thickness of the CP and VZ, was not significantly different between ADHD-derived and control-derived organoids on day 35 (*p* > 0.05), whereas the NE of ADHD-derived organoids was thinner than that of control-derived organoids on day 56 (*p* < 0.001). Moreover, although the thickness of the NE of control-derived organoids was significantly increased between day 35 and 56 (*p* < 0.001), that of ADHD-derived organoids showed no significant change.

### Thickness of CP and VZ

Next, we analyzed the thickness of the CP contained in the NE and found that the CP of ADHD-derived organoids was thinner than that of control-derived organoids on both day 35 and day 56 (day 35: *p* < 0.05; day 56: *p* < 0.001, Fig. 1d). On the other hand, VZ was not significantly different between ADHD-derived organoids and control-derived organoids on day 35 (*p* > 0.05, Fig. 1e), whereas VZ of ADHD-derived organoids was significantly thinner than control-derived organoids on day 56 (*p* < 0.01). In the comparison of the thickness between day 35 and 56, the thickness of the CP was significantly increased in both control- and ADHD-derived organoids (control: *p* < 0.001; ADHD: *p* < 0.001), whereas the VZ of ADHD-derived organoids was not significantly increased between day 35 and 56. These results suggested that the CP of ADHD-derived organoids, the cerebral cortex in early development, was thinner than that of control-derived organoids, possibly due to the alteration in the growth of the VZ, the layer of neural stem cells.

### Number of Cells in Layer VI and V of the Cerebral Cortex

**Fig. 2:**
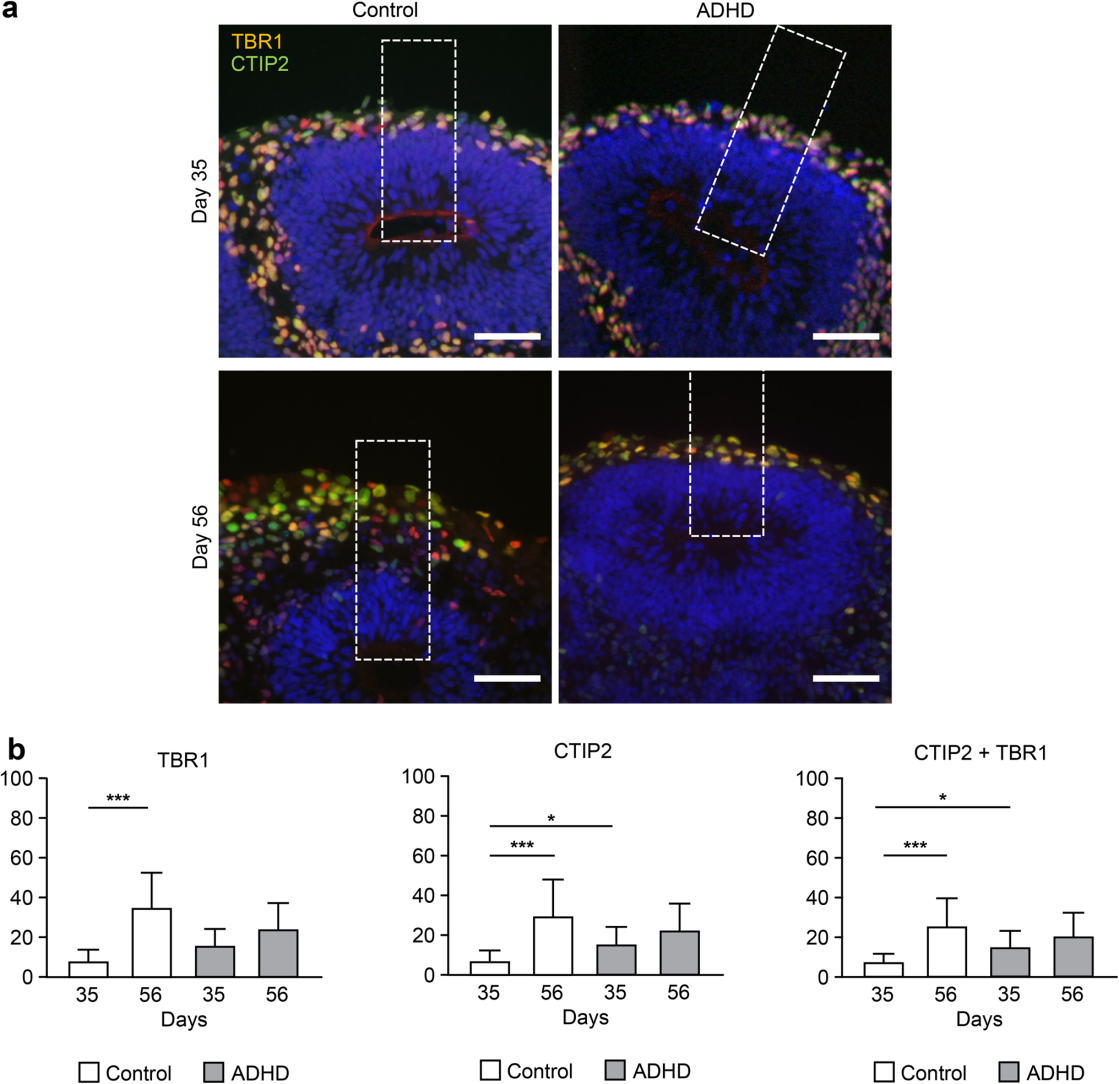
Comparison of the number of TBR1- and/or CTIP2-positive cells. (**a**) TBR1- and/or CTIP2-positive cells in layer structures. Scale bars: 40 μm. Dotted square: ROI (60 × 200 μm). (**b**) The number of TBR1- and/or CTIP2-positive cells. Control: day 35 (*n* = 21), day 56 (*n* = 24); ADHD: day 35 (*n* = 21), day 56 (*n* = 24). *P < 0.05, **P < 0.01, ***P < 0.001

For further analysis of morphological differences, we counted the number of cells expressing TBR1 and/or CTIP2, specific marker proteins of neurons in layer VI and V of the cerebral cortex (Fig. 2a). On day 35, the CP of ADHD-derived organoids contained significantly more cells expressing CTIP2 and cells coexpressing TBR1 and CTIP2 than that of control-derived organoids (CTIP2: *p* < 0.05; coexpression of TBR1 and CTIP2: *p* < 0.05, Fig. 2b), even though their thickness was thinner than that of control-derived organoids. On the other hand, the number of cells expressing TBR1 and/or CTIP2 significantly increased between day 35 and 56 in control-derived organoids (*p* < 0.001), whereas no significant increase was observed in ADHD-derived organoids. These results showed altered growth of the early cortex, meaning that an increase in the number of neurons was disrupted in ADHD-derived organoids.

### Number of Cell Divisions

**Fig. 3:**
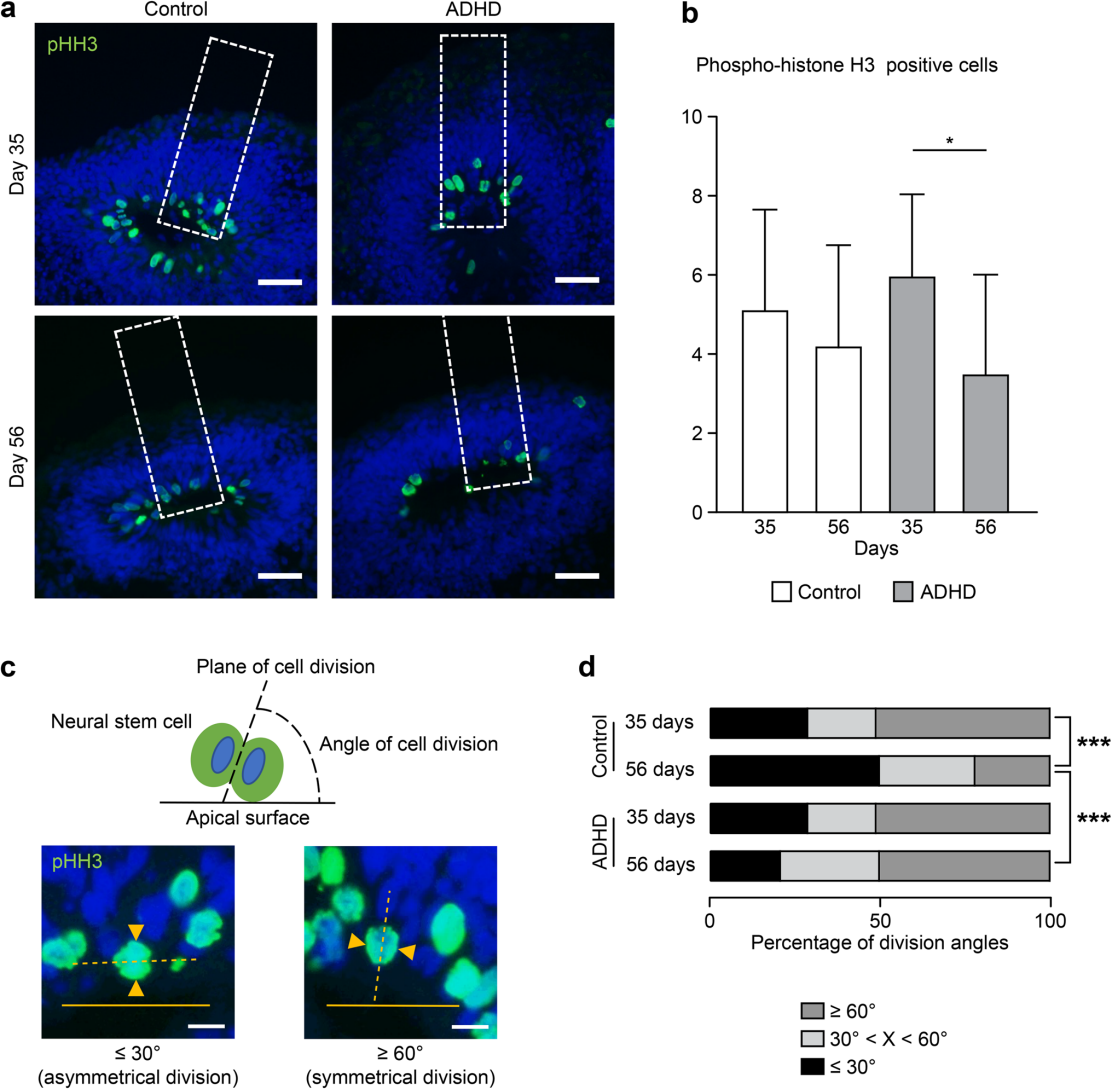
The number of cell divisions and symmetric/asymmetric divisions. (**a**) Cell division indicated by pHH3 in layer structures. Scale bars: 40 μm. Dotted square: ROI (60 × 200 μm). (**b**) The number of pHH3-positive cells. Day 35: Control (*n* = 26), ADHD (*n* = 26); day 56: Control (*n* = 9), ADHD (*n* = 9). *P < 0.05. (**c**) Examples of the angle of cell division. ≥ 60° was considered symmetric, and ≤ 30° was considered asymmetric. Scale bars: 10 μm. (**d**) Ratio of the angle of cell division. The measurement results were divided into three groups: ≥ 60° (dark gray), 30–60° (light gray), and ≤ 30° (black). day 35: Control (*n* = 65), ADHD (*n* = 65); day 56: Control (*n* = 32), ADHD (*n* = 24). ***P < 0.001

Our morphological analysis showed that ADHD-derived organoids had a thinner layer structure than control-derived organoids, even though there were more neurons in the layers than in the control-derived organoids. This suggested that alterations occurred in the neurodevelopment of ADHD-derived organoids, including division of neural stem cells, differentiation from neural stem cells into neurons, and apoptosis of the cells. For further investigation, we counted the number of cell divisions indicated by the expression of phospho-histone H3 (pHH3, Fig. 3a). In control-derived organoids, the number of cell divisions was not significantly changed between day 35 and 56 (Fig. 3b), indicating continuous proliferation of neural stem cells. Otherwise, in ADHD-derived organoids, the number of cell divisions was significantly decreased from day 35 to 56 (*p* < 0.05), suggesting less potential to proliferate.

### Proportion of Symmetric and Asymmetric Cell Division

Furthermore, we analyzed the proportion of symmetric and asymmetric cell division: cell division for the proliferation of neural stem cells and differentiation from neural stem cells into neurons [26, 27]. When the angle between the plane of cell division and the apical surface was less than or equal to 30 degrees, the cell division was considered asymmetric division, and when the angle was greater than or equal to 60 degrees, the cell division was considered symmetric division (Fig. 3c). From day 35 to day 56, only the control-derived organoids showed a significant change in the proportion of cell division (Fig. 3d, chi-square test, *p* < 0.001), with an increase in asymmetric division and a decrease in symmetric division. On day 56, the proportion of symmetric and asymmetric division showed a significant difference in control- and ADHD-derived organoids (chi-square test, *p* < 0.001): control-derived organoids showed more asymmetric division and less symmetric division than ADHD-derived organoids. These results suggested that the proportion of cell division gradually shifted from proliferation to differentiation in a relatively later stage of development in control-derived organoids but not in ADHD-derived organoids.

### Apoptosis and Proliferation of Cells

**Fig. 4:**
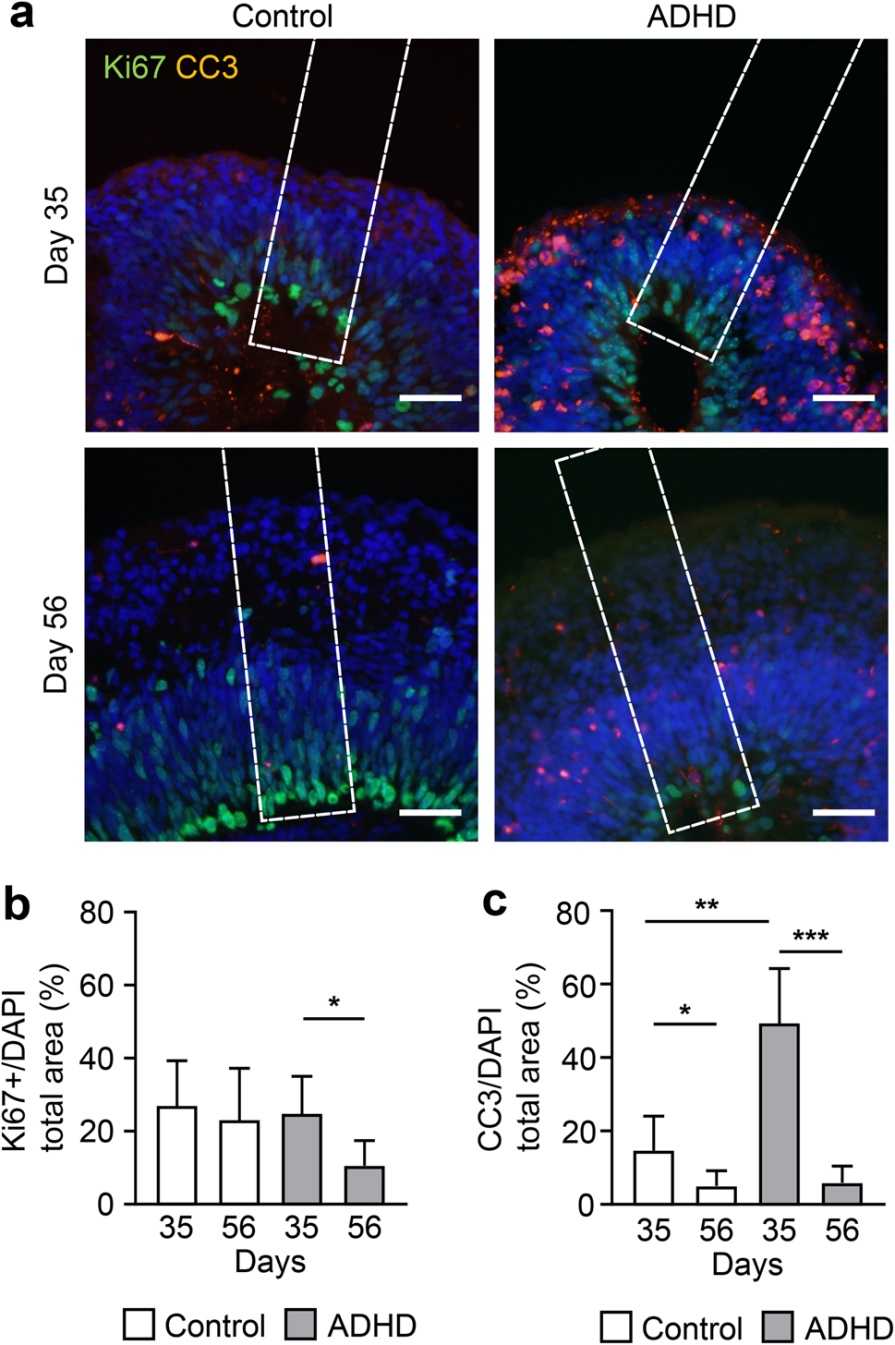
The number of cell divisions and apoptosis events. (**a**) Cell division and apoptosis indicated by Ki67 and cleaved-caspase 3 (CC3), respectively, in layer structures. Scale bars: 40 μm. Dotted square: ROI (60 × 250 μm). (**b**) Relative area of Ki67-positive cells. The Ki67-positive area was divided by the DAPI-positive area for standardization. Day 35: control (*n* = 19), ADHD (*n* = 13); day 56: control (*n* = 14), ADHD (*n* = 8). *P < 0.05. (**c**) Relative area of CC3-positive clusters. The CC3-positive area was divided by the DAPI-positive area for standardization. Day 35: control (*n* = 19), ADHD (*n* = 13); day 56: control (*n* = 14), ADHD (*n* = 8). *P < 0.05, **P < 0.01, ***P < 0.001

Finally, we distinguished possible changes in cell death. We analyzed the amount of cleaved-caspase 3 (CC3) and Ki67, the marker protein for apoptosis and proliferation of cells (Fig. 4a). In control-derived organoids, Ki67 was not significantly changed between day 35 and 56 (*p* > 0.05, Fig. 4b), consistent with the pHH3 results. On the other hand, in ADHD-derived organoids, proliferation significantly decreased between day 35 and 56. Cell death indicated by CC3 significantly decreased from day 35 to 56 in both control- and ADHD-derived organoids (control: *p* < 0.05; ADHD: *p* < 0.001, Fig. 4c), although it was significantly higher in ADHD-derived organoids than in control-derived organoids on day 35 (*p* < 0.01). These results indicated two characteristics of ADHD cells: the amount of cell proliferation was decreased in early development, and much apoptosis occurred. Alterations in proliferation and apoptosis might drive the changes in the morphology of layer structures and the number of neurons.

## Discussion

In the present study, we differentiated iPSCs derived from an ADHD individual into telencephalon organoids. During early neurodevelopment, the early cortex in the organoids had thin layer structures accompanied by poor growth of the NE and VZ. Although the CP of ADHD-derived organoids was thin, it contained more neurons than the control in the early developmental stage, and the number of neurons showed a poor increase in the following stage. ADHD-derived organoids also showed decreased cell division, an altered proportion of proliferation and differentiation, and increased apoptosis in the early developmental stage. Our biological models could reflect altered cortical neurodevelopment in ADHD-derived organoids at the same developmental stage in vivo [28–30].

Brain imaging has been used to characterize the neurobiological basis of ADHD, especially the morphology of the frontal lobes. However, the symptoms of ADHD and the delay in the morphological development of the cerebral cortex are continuously altered during maturation [17, 31]. The reduced brain size and correlated structural alterations in ADHD patients are closely related to apoptosis and neurodevelopmental genes [32]. The investigation of neurons and synaptic connections existing in the cortex has inherent limitations in imaging studies and cannot be observed in the mature brain [33, 34]. Cerebral organoids have been shown to mimic the clinical conditions of brain alteration in infants and children, as well as the differentiation process of human cortical neurons during fetal development, with advantages in the resolution of pathological development [35–37]. Our study demonstrated a biological model of ADHD reflecting early neurodevelopment, which cannot be observed in previous studies. It could contribute to providing a foundation for further research.

The regulatory effects of ADHD susceptibility genes on neural differentiation are mainly manifested in neuronal proliferation, migration, synaptic formation and synaptic plasticity [38, 39]. Regarding the differences in neural differentiation in ADHD patients, the present study found that our ADHD model contained more neurons in the CP and showed more apoptosis than the control, indicating that both more generation and more death of neurons occurred in ADHD-derived organoids. These results suggest that the neurons of ADHD patients might be more fragile than those of controls, and/or differentiation from neural stem cells to neurons might occur too early and immature neurons are generated.

Another finding we would like to discuss is the small size of neurons in ADHD, which was indicated by the findings that the CP of ADHD-derived organoids contained more neurons in the thinner layer than those in control-derived organoids. Neurodevelopmental abnormalities in the prefrontal cortex of animal models of ADHD, such as fewer branch points and roots, shorter neurite length in spontaneously hypertensive rats [40–42], and lower spine density of the pyramidal neurons in dopamine transporter knockout mice, which we reported previously [43]. It is reasonable to speculate that our results regarding the NE thickness and the number of neurons observed reflect those morphological alterations of neurons in ADHD patients.

Another important finding is the alteration in the proportion of proliferation and differentiation of neural stem cells, which play a crucial role in cortex development. Neural stem cells renew themselves and differentiate into neurons through symmetric and asymmetric cell division, and this proper balance maintains increase and growth of neurons [44–46]. Neural stem cells increase their numbers through symmetric cell division, followed by switching to asymmetric cell division to differentiate into neurons, thus controlling the correct formation of the cerebral cortex [47, 48]. Our results showed that the three-dimensional telencephalon organoid models we derived from the ADHD patient did not show a similar shift from symmetric cell division to asymmetric cell division as the control, possibly reflecting less potential to proliferate and to shift to differentiation.

These findings may be limited in part because the three iPSC lines that we used to differentiate into the organoids were derived from the same individual. Samples derived from more individuals will provide more possibilities for the establishment and comparison of organoids and can also expand the pathological analysis of ADHD.

Our in vitro models demonstrate the possible contribution of neural proliferation and differentiation and apoptosis in the formation of the early cortex and suggest the possible mechanisms of delayed cortical maturation in ADHD patients. These results show the alterations in the characteristics of neural stem cells and the formation of layer structures, potentially indicating key players in the pathogenesis of ADHD.

## Supplementary Information

Supplementary Fig. 1. Induction of iPS cells from an ADHD patient. The three iPS cell lines (ADHD-1,2,3) were generated from the same patient. (a) Immunocytochemistry for Nanog, OCT3/4, SOX2, SSEA4, and TRA-1-60. Scale bars: 100 μm. (b) Embryoid body–mediated differentiation of human iPS cells. Immunocytochemistry of TUJ1, α-smooth muscle actin (α-SMA) and α-fetoprotein (AFP). Scale bars: 200 μm (PNG 1148 kb)

High resolution image (TIF 7.92 MB)

Supplementary Fig. 2. Cytogenetic analysis of the cultured iPS cells by Q-banding (PNG 856 kb)

High resolution image (TIF 3.25 MB)

Supplementary Fig. 3. Time schedule of telencephalon organoid generation (PNG 192 kb)

High resolution image (TIF 1.05 MB)

## Data Availability

The datasets generated and/or analyzed during the current study are available from the corresponding author on reasonable request.
