# Supplementary figures and images for: Telencephalon Organoids Derived from an Individual with ADHD Show Altered Neurodevelopment of Early Cortical Layer Structure
Source: Stem Cell Rev Rep. 2023 Mar 6;19(5):1482–91. doi: 10.1007/s12015-023-10519-z (PMC10366301; doi:10.1007/s12015-023-10519-z)

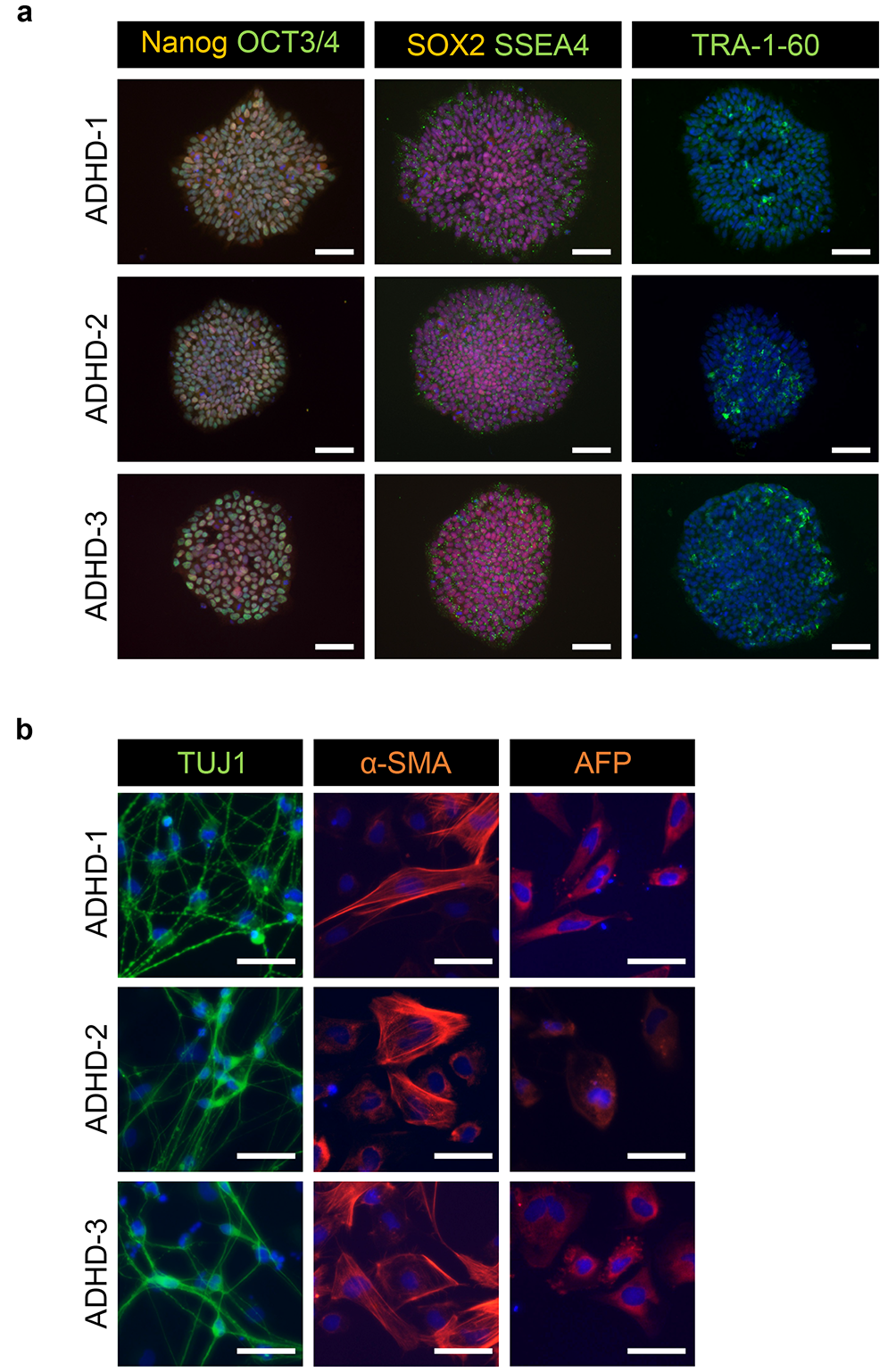

Supplement: Supplementary file 1 — Induction of iPS cells from an ADHD patient. The three iPS cell lines (ADHD-1,2,3) were generated from the same patient. (a) Immunocytochemistry for Nanog, OCT3/4, SOX2, SSEA4, and TRA-1-60. Scale bars: 100 μm. (b) Embryoid body–mediated differentiation of human iPS cells. Immunocytochemistry of TUJ1, α-smooth muscle actin (α-SMA) and α-fetoprotein (AFP). Scale bars: 200 μm (PNG 1148 kb) [file 12015_2023_10519_Fig5_ESM.png]

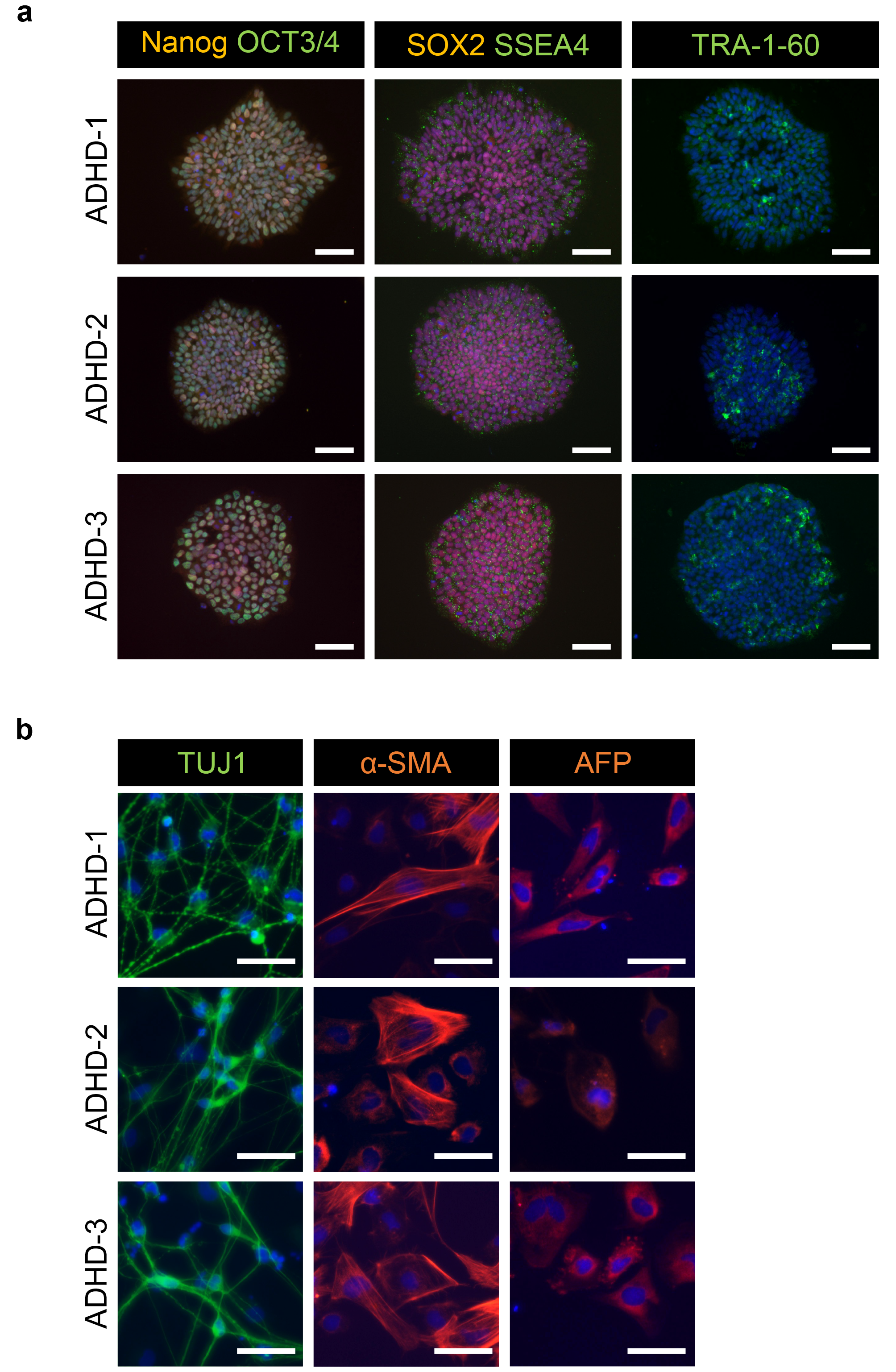

Supplement: Supplementary file 2 — High resolution image (TIF 7.92 MB) [file 12015_2023_10519_MOESM1_ESM.tif]

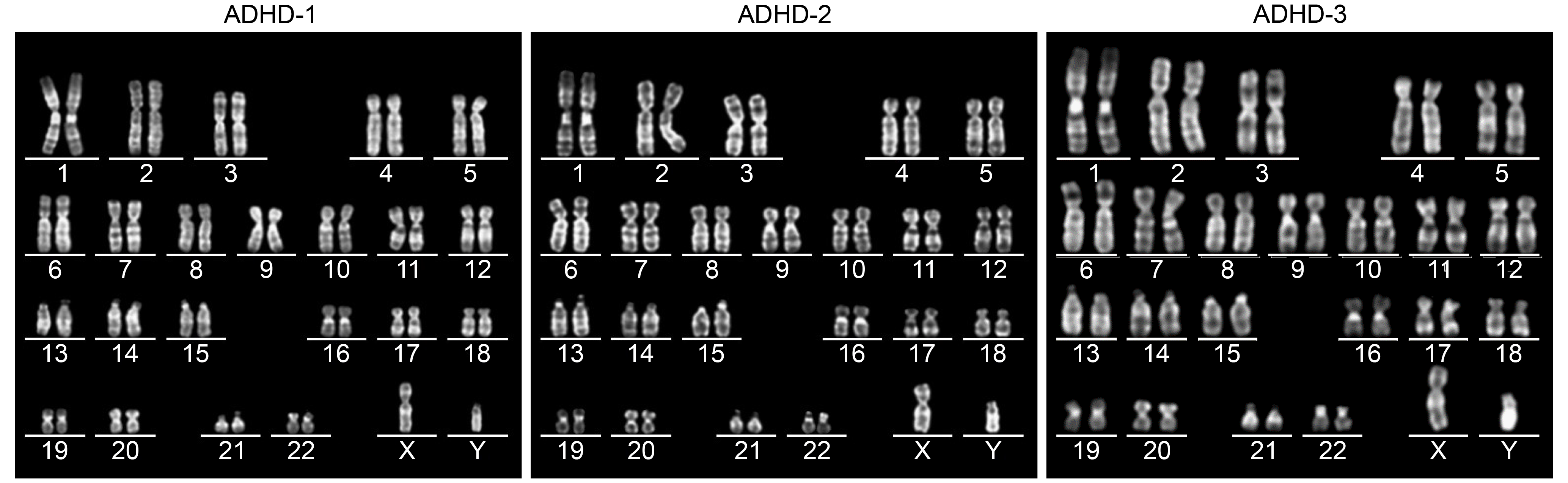

Supplement: Supplementary file 3 — Cytogenetic analysis of the cultured iPS cells by Q-banding (PNG 856 kb) [file 12015_2023_10519_Fig6_ESM.png]

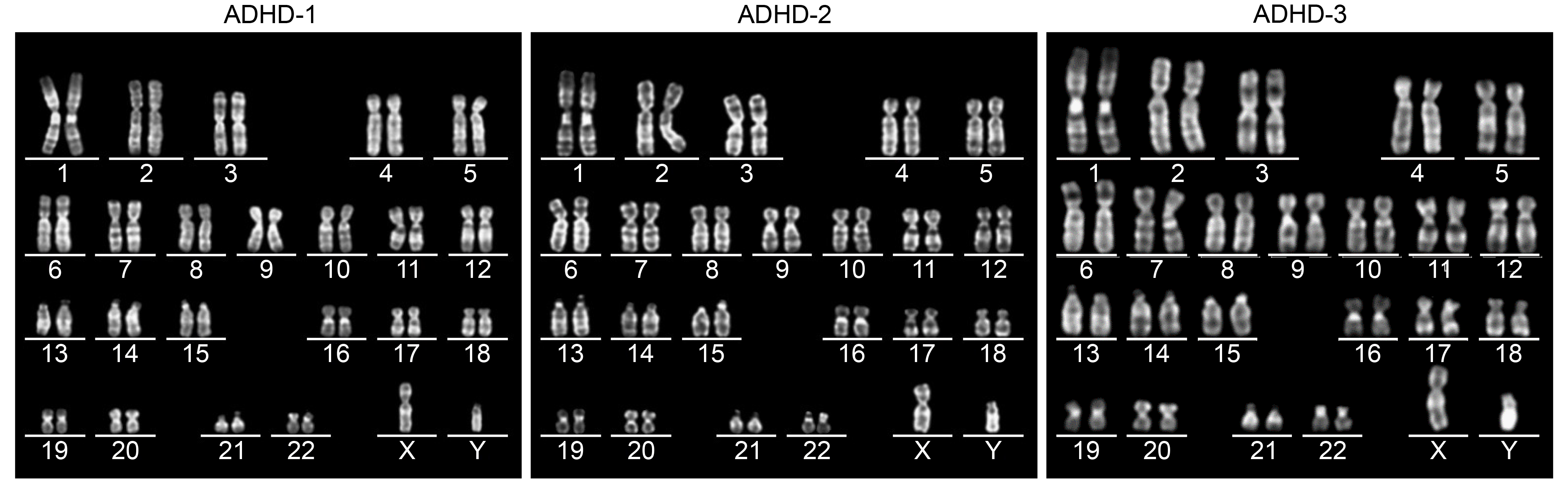

Supplement: Supplementary file 4 — High resolution image (TIF 3.25 MB) [file 12015_2023_10519_MOESM2_ESM.tif]

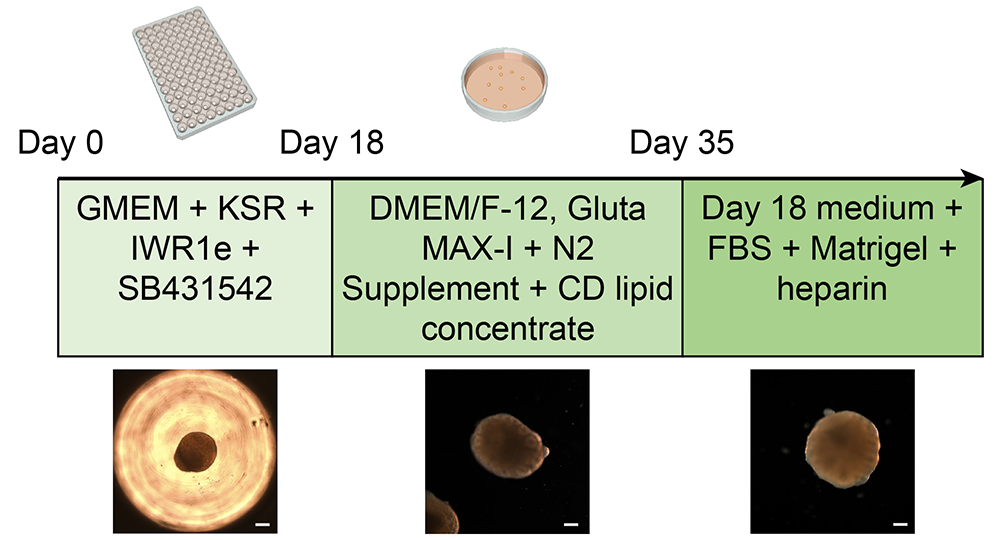

Supplement: Supplementary file 5 — Time schedule of telencephalon organoid generation (PNG 192 kb) [file 12015_2023_10519_Fig7_ESM.png]

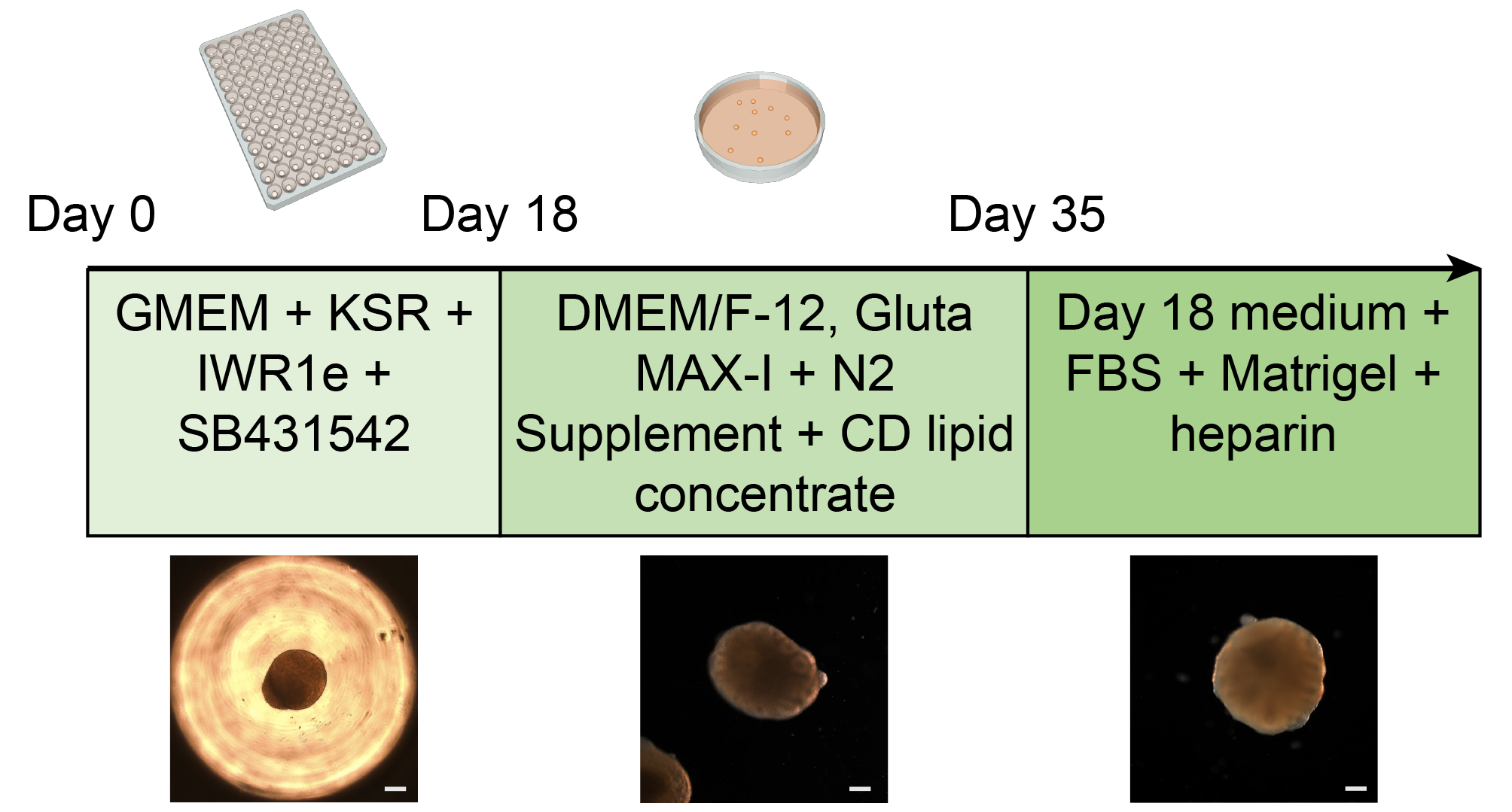

Supplement: Supplementary file 6 — High resolution image (TIF 1.05 MB) [file 12015_2023_10519_MOESM3_ESM.tif]
